# Metabolic Abnormalities of Erythrocytes as a Risk Factor for Alzheimer's Disease

**DOI:** 10.3389/fnins.2017.00728

**Published:** 2018-01-05

**Authors:** Elena A. Kosenko, Lyudmila A. Tikhonova, Carmina Montoliu, George E. Barreto, Gjumrakch Aliev, Yury G. Kaminsky

**Affiliations:** ^1^Institute of Theoretical and Experimental Biophysics, Russian Academy of Sciences, Pushchino, Russia; ^2^Fundación Investigación Hospital Clínico, INCLIVA Instituto Investigación Sanitaria, Valencia, Spain; ^3^Departamento de Nutrición y Bioquímica, Facultad de Ciencias, Pontificia Universidad Javeriana, Bogotá, Colombia; ^4^Instituto de Ciencias Biomédicas, Universidad Autónoma de Chile, Santiago, Chile; ^5^GALLY International Biomedical Research Institute Inc., San Antonio, TX, United States

**Keywords:** Alzheimer's disease, amyloid β, erythrocytes, metabolic dysfunction, multilayered pathology, clinical manifestation

## Abstract

Alzheimer's disease (AD) is a slowly progressive, neurodegenerative disorder of uncertain etiology. According to the amyloid cascade hypothesis, accumulation of non-soluble amyloid β peptides (Aβ) in the Central Nervous System (CNS) is the primary cause initiating a pathogenic cascade leading to the complex multilayered pathology and clinical manifestation of the disease. It is, therefore, not surprising that the search for mechanisms underlying cognitive changes observed in AD has focused exclusively on the brain and Aβ-inducing synaptic and dendritic loss, oxidative stress, and neuronal death. However, since Aβ depositions were found in normal non-demented elderly people and in many other pathological conditions, the amyloid cascade hypothesis was modified to claim that intraneuronal accumulation of soluble Aβ oligomers, rather than monomer or insoluble amyloid fibrils, is the first step of a fatal cascade in AD. Since a characteristic reduction of cerebral perfusion and energy metabolism occurs in patients with AD it is suggested that capillary distortions commonly found in AD brain elicit hemodynamic changes that alter the delivery and transport of essential nutrients, particularly glucose and oxygen to neuronal and glial cells. Another important factor in tissue oxygenation is the ability of erythrocytes (red blood cells, RBC) to transport and deliver oxygen to tissues, which are first of all dependent on the RBC antioxidant and energy metabolism, which finally regulates the oxygen affinity of hemoglobin. In the present review, we consider the possibility that metabolic and antioxidant defense alterations in the circulating erythrocyte population can influence oxygen delivery to the brain, and that these changes might be a primary mechanism triggering the glucose metabolism disturbance resulting in neurobiological changes observed in the AD brain, possibly related to impaired cognitive function. We also discuss the possibility of using erythrocyte biochemical aberrations as potential tools that will help identify a risk factor for AD.

## Introduction

Alzheimer's disease (AD) is a slowly progressing, systemic neurodegenerative disorder of uncertain etiology. Clinical manifestation of this disorder usually consists of cognitive deficits in memory in the elderly. Some estimates suggest that 50% of the population over the age of 80 years suffers from this type of dementia. With increases in life expectancy of our population, AD is already approaching epidemic proportions with no cure or preventative therapy yet available. Now, AD affects ~24 million people worldwide with 4.6 million new cases of dementia every year (one new case every 7 s), and if existing trends continue, 115 million individuals worldwide will have Alzheimer's disease (AD) by 2050 (Wimo and Prince, 2010; Fita et al., 2011).

AD develops sporadically in 95–98% of the AD population (Bird, 2005; Reddy, 2006; Kaminsky et al., 2010). However, the genetic-linked cases have provided a great deal of biochemical insights in the disease process. The research field has been focused on the role of Aβ in the brain stemming from the fact that accumulation of these peptides results in aggregation and formation of insoluble plaques, which trigger a cascade of deleterious changes, leading to neuronal death and thus causing AD. This train of events has been called the amyloid-cascade hypothesis of AD (Hardy and Higgins, 1992). It is significant that accumulation of aggregated Aβ is the primary abnormality in AD and that its deposition is required for postmortem diagnosis. Now, however, a large body of evidence exists, and new data continues to accumulate indicating that the number of Aβ deposits in the brain does not correlate well with the degree of cognitive impairment (Braak and Braak, 1991; Terry et al., 1991; Giannakopoulos et al., 2003; Guillozet et al., 2003). Indeed, Aβ deposition may occur in normal non-demented elderly people (Joachim et al., 1989; Mann et al., 1992; Lue et al., 1999; Schmitt et al., 2000; Pike et al., 2007), that is in agree with the fact that virtually all humans start to accumulate Aβ in the brain upon aging (Funato et al., 1998; Wang et al., 1999; Morishima-Kawashima et al., 2000). Besides, amyloid plaques are not specific for Alzheimer's disease and have been found in many other pathological conditions, including transmissible spongiform encephalopathies (Liberski, 2004), Down's syndrome (Glenner and Wong, 1984), Lewy body in Parkinson's disease (Arai et al., 1992), acute traumatic brain injury with diffuse axonal damage (Smith et al., 2003) and chronic traumatic brain injury associated with boxing (Roberts et al., 1990; Jordan, 2000) and football (Omalu et al., 2005). What is clear from these studies is that the presence of brain plaques alone is insufficient to produce cognitive decline in AD (Jack et al., 2009) and that such studies support the basis for the formation of a new hypothesis.

Recently, a modified Aβ-cascade hypothesis has been formulated that predicts intraneuronal accumulation of soluble Aβ oligomers, but not monomer or insoluble amyloid fibrils, as the first step of a fatal cascade in AD (McLean et al., 1999; Wirths et al., 2004; Selkoe, 2007). The amyloid oligomerization is observed to occur intracellularly (Connolly and Volpe, 1990) and Aβ_1−42_ oligomers turn out to be potent neurotoxins in animal brain and neuronal cultures where they are able to disrupt glutamatergic synaptic function (Lambert et al., 1998; Hsia et al., 1999; Klein et al., 2001; Hardy and Selkoe, 2002; Kamenetz et al., 2003; Walsh and Selkoe, 2004; Roselli et al., 2005) and neuronal calcium homeostasis (Bapat et al., 1983; Mattson et al., 1992; Demuro et al., 2005), promote abnormal release of glutamate in hippocampal neurons (Brito-Moreira et al., 2011), induce oxidative stress (De Felice et al., 2007), incite tau hyperphosphorylation (De Felice et al., 2008), and synapse loss (Lue et al., 1999; Selkoe, 2008), inhibit long-term potentiation in the hippocampus (Walsh et al., 2002), which is required for memory formation, and in turn leads to the cognitive deficits in the animal. Using oligomer-sensitive immunoassay, the soluble Aβ oligomers have been found in brains of AD patients (Kuo et al., 1996; Lue et al., 1999; Gong et al., 2003). This confirms the prediction that soluble oligomeric Aβ-forms are characteristic of AD pathology. However, the soluble Aβ burden displayed considerable individual variation in the brain of AD patients. Thus, the mean level of soluble Aβ can increase 3-fold (McLean et al., 1999), 6-fold, 12-fold (Kuo et al., 1996), and 70-fold (Gong et al., 2003) in brain of AD patients compared to age-matched control, at that, the majority of soluble peptides was Aβ_1−42_ (Kuo et al., 1996). On the other hand, it was found that the levels of soluble Aβ_1−42_ were smallest in the AD brain (0.7%) and that the soluble pools of Aβ_1−40_ and Aβ_1−42_ were the largest fractions of total Aβ in the normal brain (50 and 23% respectively, Wang et al., 1999). Other authors also showed that the Aβ_1−42_ levels were found in the brains of normal elderly subjects (Tabaton and Piccini, 2005) and that in subjects with AD these concentrations increased slightly compared with the age-matched control (Lue et al., 1999). These studies suggest that within individual AD subjects, the areas with greater numbers of soluble Aβ oligomers did not, as a rule, and whether the levels of these “concentration-jumping” oligomers correlate with the memory decline in AD remains to be determined. Indeed, previous studies have shown that these Aβ forms were observed in the brains of patients with Down's syndrome (DS) (Teller et al., 1996; Gyure et al., 2001; Tabaton and Gambetti, 2006) indicating that the accumulation of soluble oligomers are not specific for AD. Moreover, in brains of patients with DS, increased levels of oxidative damage occur prior to the onset of Aβ deposition (Nunomura et al., 2000). Hence, the formation of diffuse amyloid plaques may be considered as the message talking about the disruption of brain homeostasis or as a compensatory response to remove reactive oxygen species (Atwood et al., 2003). Thus, these facts provide the opportunity to investigate the pathological conditions that precede the formation of the Aβ deposits in the human brain.

It is well known that a characteristic reduction of cerebral perfusion and metabolism occurs in patients with AD (de la Torre, 2000b; Aliev et al., 2003a). It was suggested that capillary distortions commonly found in the AD brain elicit hemorheological changes that altered the delivery and transport of essential nutrients, particularly glucose, and oxygen required for its aerobic oxidation in brain cells (de la Torre and Mussivand, 1993; de la Torre, 2002a; Chang et al., 2007; Aliev, 2011) resulting in an energy metabolic breakdown of the biosynthetic and synaptic pathways, subsequently leading to the death of neurons as a consequence of cognitive deterioration. In fact, it was proposed that AD may originate as a vascular disorder with the resultant impairment of oxygen delivery to the brain with the plaques and tangles found in the brain secondary to the effects of the vascular pathology (de la Torre, 2002a). Another important factor in tissue oxygenation is the ability of red blood cells (RBC) to the binding, transport and delivery of oxygen to tissues that depends, first of all, on RBC energy metabolism and antioxidant status (Brewer et al., 1974) that is extremely important for the functioning and regulation of oxygen affinity to hemoglobin (van Wijk and van Solinge, 2005).

Surprisingly, despite the main role of RBC metabolism in the delivery of oxygen to the tissues, no systematic programs of research have examined the relationship between the breach of the energy metabolism of these cells in destabilization of glucose metabolism in the brain pathology and this relationship is still not sufficiently discussed in the literature. Therefore, our current hypothesis is that RBC metabolism plays a key role in AD brain disorders. We propose that the long-term lack of sufficient energy, disturbance of glycolytic, antioxidant RBSs pathways, and sodium potassium pump in oldest subjects [caused by different reasons and also in contact with Aβ, which is located on the luminal surfaces of cerebral microvessels (Grammas et al., 2002; Michaud et al., 2013)] can cause a decrease in the ability of RBC to transfer oxygen to tissue, leading to inadequate oxygenation and can result in abnormal glucose/energy metabolism, oxidative stress and, thereby, increase the susceptibility of neurons to damage, and reduce mental capacity as a consequence thereof. We have called this chain of events as “*the erythrocytic hypothesis of Alzheimer disease”* (Kosenko et al., 2016). In support of this hypothesis we also believe that erythrocyte biochemical aberrations might be used as potential tools in the early detection of the brain pathology development. This hypothesis provides ideas for the development of innovative personalized medical technologies allowing recovering the energy metabolism and the system of antioxidant defense in erythrocytes.

## Brain glucose metabolism, glutamate toxicity, and Aβ accumulation: cause or effect?

The brain is normally dependent on glucose for oxidative metabolism and function, therefore it is extremely sensitive to fluctuation in the blood glucose concentration, and since no satisfactory brain endogenous substitute exists. In spite of the fact that under certain conditions such as starvation or diabetes the ketone bodies can supply up to 50% of the brain's energy needs, the rest of the energy anyway must come from glucose. Therefore, within even just a few minutes glucose and oxygen deprivation induces significant dysfunction, and a longer time period can ultimately result in cell death (Blass, 2002). In addition to ATP production, the oxidation of glucose can produce other important intermediate such as lactate, which does not enter necessarily in the tricarboxylic acid cycle, but rather can be released and transported by the circulation into the liver for glucose synthesis de novo. Glucose also can be incorporated into lipids, proteins, and glycogen, and it is also the precursor of certain neurotransmitters such as γ-aminobutyric acid (GABA) (Plum and Posner, 1972), glutamate (Hamberger et al., 1979), and acetylcholine (Gibson et al., 1975). Thus, circulating glucose regulates many brain functions, including brain vitality, activity, learning, and memory (Korol and Gold, 1998).

Whereas the cerebral energy status is only slightly decreased during the normal aging process, glucose metabolism, and cellular ATP production are severely reduced in sporadic AD (Kyles et al., 1993; Hoyer, 1996). Certain neuronal populations are especially vulnerable to cut glucose oxidation, specifically neurons in the CA1, subiculum, and dentate gyrus of the hippocampus, and neurons in the outer layers of the cortex (Auer and Siesjö, 1993). A substantial proportion of neurons in these regions is glutamatergic and evidence suggests that hypoglycemic injury in these neurons is initiated almost entirely by hyperactivation of glutamate receptor (Auer et al., 1985), followed by the glutamate cascade and oxidative stress. The numerous studies have provided conclusive proof that glutamate becomes neurotoxic via the NMDA receptor when intracellular energy levels are reduced (Novelli et al., 1988; Beal et al., 1991; Albin and Greenamyre, 1992; Beal, 1992; Storey et al., 1992; Kosenko et al., 1994; Gonzalez et al., 2015). On the other hand, there is a direct relationship between disturbances in energy metabolism and mental disorder. For example, in 1932 Quastel J. first put forward a general suggestion that disturbances in energy metabolism would impair the neurological function, including particularly cognition (Quastel, 1932). During the past decades, a lot of work has proved Quastel's theory to be prescient and showed that the cause-effect relation is nonspecific as impairing cerebral energy metabolism can induce mental disorders to varying degrees (confusion, mental fatigue, agnosia, or dementia) in different pathological situations. Thus, impaired mental function has been reported in association with hypoglycemia (Bruce et al., 2009), inadequate transportation of glucose across the blood-brain barrier (Klepper and Voit, 2002; Pascual et al., 2004), defective astroglial glutamate transportation (Rönnbäck and Hansson, 2004), hypoxia (Gibson et al., 1981), diabetes (Richardson, 1990), heart failure (Riegel et al., 2002), reduced glucose tolerance (Vanhanen et al., 1997), bradycardia, hypotension (Ackerman, 1974), high intracranial pressure (Yoshida et al., 1996), stroke (van der Zwaluw et al., 2011), hypothermia, alcohol intoxication, thiamine and vitamin C deficiency, sedative-hypnotic drugs, opioids consumption (Martindale et al., 2010), general anesthesia (Parikh and Chung, 1995; Xie et al., 2006b), hypocapnia (Dodds and Allison, 1998), chronic stress (Conrad et al., 1996; Conrad, 2006), chronic noise stress (Arnsten and Goldman-Rakic, 1998; Manikandan et al., 2006), mixed brain pathologies (Schneider et al., 2007), hepatic encephalopathy (Butterworth, 2003), hyperammonemia (Llansola et al., 2007), trauma (Brooks et al., 2000), and so forth. Interestingly, after trauma, a large number of Aβ positive neurons appeared in human (Chen et al., 2004; Uryu et al., 2007) and animal brain (Kamal et al., 2001; Kasa et al., 2001; Papp et al., 2002; Hamberger et al., 2003). APP (amyloid precursor protein) accumulation is also observed following rat (Li et al., 1995) and human spinal cord injury (Ahlgren et al., 1996; Cornish et al., 2000). Long-term presence of APP and accumulation of Aβ in the rat thalamus were observed after middle cerebral artery occlusion (van Groen et al., 2005) and in cultured cells that had been treated with spirochetes or bacterial lipopolysaccharide (LPS) (Miklossy et al., 2006) and other infectious agents (Balin and Appelt, 2001).

A number of studies have also demonstrated that abnormal activation of β-adrenergic receptors (β-ARs), which mediate the effect of stress, might contribute to Aβ peptides production resulting in accelerating amyloid plaque formation *in vitro* and *in vivo* by enhancing γ-secretase activity (Ni et al., 2006) and that blocking β-ARs attenuates acute stress-induced Aβ peptide production (Yu et al., 2010). Indeed, the common inhalation anesthetic isoflurane has been reported to increase brain Aβ protein levels *in vitro* (Xie et al., 2006a) and *in vivo* (Xie et al., 2008; Zhang et al., 2008; Dong et al., 2009). Hypocapnia can also increase Aβ production in H4 human neuroglioma cells (Xie et al., 2004). Nanoscale particulates, a major component airborne pollution, inducing the blood-brain barrier disruption and neuroinflammation (Murr et al., 2006), result in AD-associated Aβ_1−42_, accumulation in the brains of children living in the high-pollution area (Calderón-Garcidueñas et al., 2008a,b). Upon careful analysis of these pathologies, one can see that there is a steady disruption of brain aerobic metabolism and the subsequent increase in APP processing and the formation of amyloids (Gabuzda et al., 1994; Webster et al., 1998; Velliquette et al., 2005). Thus, according to positron emission tomography (PET), isoflurane anesthesia can cause a 50% decrease in the rate of glucose uptake by the brain (Alkire et al., 1995, 1997), which leads to a sharp inhibition of aerobic oxidation in the cells and development of severe hypoxia, decreased neuronal activity (Hodes et al., 1985), and the appearance of amyloid in the brain 6-24 h after application of the anesthetic (Xie et al., 2006a, 2008). In ischemia-reperfusion, in addition to increasing oxidative stress, there is a decrease in the rate of blood flow, since migration of neutrophils to the site damaged by hypoxia can cause blockage of capillaries (Simpson et al., 1988), which impairs the entry of glucose and oxygen into the brain and promotes the formation of amyloids in damaged brain structures (van Groen et al., 2005; Tesco et al., 2007). In hypoglycemia, the limited supply of glucose from the blood to the brain also contributes to the accumulation of amyloids in the brain (Shi et al., 1997).

Altogether, these findings suggest that a transient insult, e.g., trauma, ischemia, neuroinflammation, anesthesia, or infectious agents could lead to secondary and persistent brain injuries and that the initial production of Aβ and its precursor, perhaps, are associated with physiological compensatory mechanisms for repair or protection of neurons exposed to significant disturbances in homeostasis (Smith et al., 2000; Lee et al., 2004). These facts are consistent with the numerous data showing that amyloid exhibits trophic and neuroprotective (Whitson et al., 1989; Koo et al., 1993; Singh et al., 1994; Luo et al., 1996), antioxidant (Smith et al., 1998, 2002a; Kontush et al., 2001; Atwood et al., 2002) properties and accumulates in the tissue after impairment of the energy metabolism with non-specific stimulus (Gabuzda et al., 1994; Webster et al., 1998; Velliquette et al., 2005), while under physiological conditions the diurnal fluctuation of brain Aβ levels is strictly regulated (Kang et al., 2009). Additionally, scores obtained on mini-mental state examination in AD subjects correlate highly with reductions of glucose metabolism (Blass, 2003), suggesting that the metabolic lesion precedes the development of neuropsychological abnormalities (Gibson and Huang, 2002) and support the conclusion that sporadic AD is a hypometabolic disorder which is provoked by a dysfunctional cerebral energy metabolism (Hoyer et al., 1988; Blass and Gibson, 1991; Meier-Ruge and Bertoni-Freddari, 1996; Perry et al., 1998; Smith et al., 2002b; Aliev et al., 2003b, 2004; Zhu et al., 2007). Obviously, the detection of mechanisms of disturbance of aerobic glucose metabolism in the brain is one of the most pressing tasks which will facilitate further progress on to determine not only to the midlife AD risk factor, but also on the lifespan of the older persons. Therefore, any pharmacological intervention, directed at correcting the chronic hypoperfusion state would possibly change the natural course of development of dementing neurodegeneration (Aliev et al., 2003a).

## The possible role of RBC in pathogenesis of AD

The pathologic causes of brain glucose metabolism disorders in AD may vary in signs and symptoms, which are as follows: desensitization of the neuronal insulin receptor (Hoyer, 2000), a decrease in the enzymes of the tricarbonic acid cycle activities (Meier-Ruge et al., 1984; Marcus et al., 1989; Marcus and Freedman, 1997; Bubber et al., 2005), impaired glucose transporter at the blood-brain barrier (Kalaria and Harik, 1989), depressed glucose transport into neurons (Simpson and Davies, 1994; Simpson et al., 1994), hippocampal region atrophy (Jobst et al., 1992; Villain et al., 2008) neuronal loss in the affected areas (McGeer et al., 1990), NO-dependent endothelial dysfunction and degeneration (De Jong et al., 1999; de la Torre, 2000a, 2002b) in brain capillaries that affect the capillary blood flow and optimal delivery of glucose and oxygen to neuronal cells (de la Torre, 2000b; Aliev et al., 2003a).

**Figure 1:**
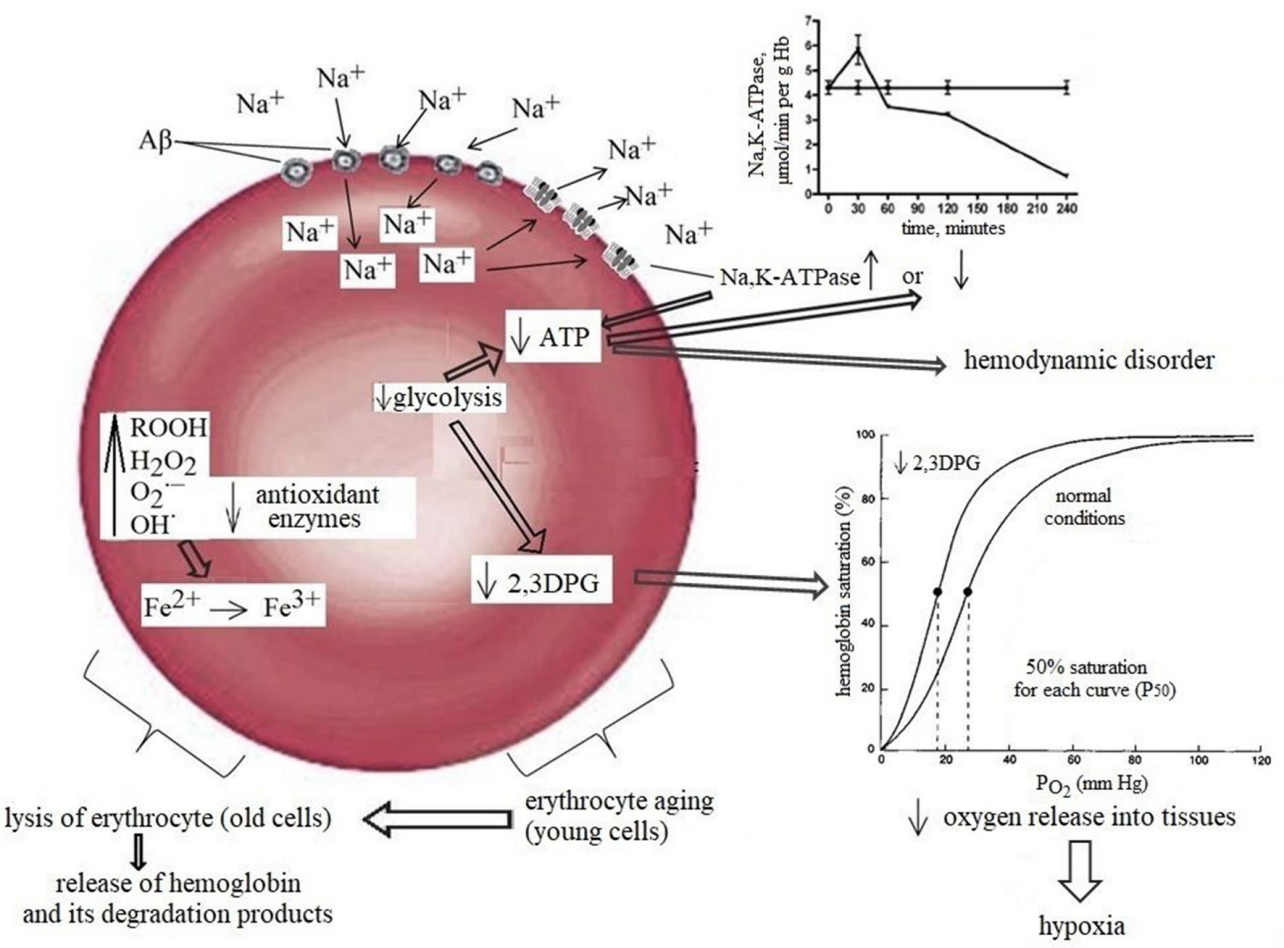
Normal aging diminishes RBC functions, including a detectable decrease in the activity of glycolytic and antioxidant enzymes. The combined effects of these damage together with a slight but significant decrease in 2,3-DPG are most likely contributor to the morphological changes in oldster subject which may result in decreased erythrocyte deformability, alter rheology, loss of adequate oxygen delivery and reduce the threshold for the development of neuropathology. The left part of the scheme: Amyloids possess gramicidin D-like action and upon contact with erythrocytes rapidly increase the concentration of sodium in the cells causing rapid activation of the Na^+^, K^+^-ATPase leading to the increase in ATP and 2,3DPG hydrolysis and can increase in Hb affinity to oxygen, that may be one of the factors contributing to brain hypoxia which lead to glucose hypometabolism and memory dysfunction in AD. The right part of the scheme: Prolonged contact with erythrocytes depletes ATP stores, causing Na^+^, K^+^-ATPase pumps and Na^+^- dependent ion channels to stop working and, consequently, the erythrocytes to swell and lyse. RBCs release hemoglobin, which is a source of iron. In turn, this metal catalyses the formation of toxic reactive oxygen species that mediate neuronal injury.

Another important factor in tissue oxygenation is the ability of RBC to bind, transport and release oxygen to tissues. For this, the RBC requires several essential metabolic pathways such as (i) anaerobic glycolysis, which is the only source of energy (ATP production) for sustaining cell structure and function; (ii) maintenance of the electrolyte gradient between plasma and red cell cytoplasm through the activity of adenosine triphosphate (ATP)-driven membrane pumps; (iii) pentose phosphate shunt (PPS) that controls the anti-oxidant pathways by produced NADPH, which plays an important role in maintaining glutathione in the reduced state; (iv) antioxidant pathways necessary for the protection of RBC proteins and hemoglobin against oxidation; and (v) nucleotide metabolism for the maintenance of the purine and pyrimidine homeostasis. Moreover, erythrocytes possess a unique glycolytic bypass, Rapoport-Luebering shunt to produce 2,3-diphosphoglycerate (2,3-DPG), a crucial metabolite in the regulation of hemoglobin affinity for oxygen (Cho et al., 2008). Thus, the mature erythrocyte retains a strictly regulated system of soluble enzymes, structural proteins, carbohydrates, lipids, anions, cations, cofactors, metabolites, antioxidants all of which are required in balance for effective metabolism and functioning of the cell. A change of at least one component of this system will lead to an imbalance and loss of RBC functional capacity. Indeed, a significant loss in ATP (Rabini et al., 1997), Mg^2+^, Na^+^, and ATP-ase activity (Ajmani and Rifkind, 1998) all of which may decrease erythrocyte deformability (Sakuta, 1981; Kucukatay et al., 2009), changes morphology (Gov and Safran, 2005) and increases RBC volume (Kowluru et al., 1989; Kucukatay et al., 2009). Extensive diminution of intracellular antioxidant GSH promotes oxidative damage of protein and lipids and compromises structural integrity of the RBC (Morris et al., 2008). Decreased 2,3-DPG, operating as a regulator of the oxygen affinity of RBC (Duhm, 1971) reduces the ability of RBC to release oxygen, resulting in tissue hypoxia (MacDonald, 1977; Nakamura et al., 1995; Figure 1). Considering the cause-effect relationship between various intracellular metabolic pathways and RBC function, it may be inferred that intact biochemical intracellular pathways are a major factor controlling the paramount RBC function associated with the ability to bind, transport, and release oxygen to tissues.

Recently, we measured some parameters of adenine nucleotide metabolism, glycolysis, pentose phosphate pathway, 2,3-DPG shunt (Kaminsky et al., 2013), oxidant and antioxidant enzymes and metabolites (Kosenko et al., 2012) in RBCs samples from Alzheimer's subjects (AD) and non-Alzheimer's dementia (NA) patients. We found that activities of all glycolytic, pentose phosphate pathway and 2,3-DPG shunt enzymes, Na^+^, K^+^-ATPase, as well as NAD/NADH ratio, pyruvate and lactate levels evidently decreased in aging and increased equally in AD and NA to levels or above levels of the YC (young controls) group indicating an increase in RBC glycolysis and ion fluxes. Elevated Na^+^, K^+^-ATPase activity and decreased ATP levels imply that ATP loss was mostly based on energy-expending redistribution of Na^+^ and K^+^ across the plasma membrane in erythrocytes from AD patients. These results confirm the fact that in AD, as in certain other diseases the balance between ATP formation and ion pumping may be disordered resulting in a decrease in intercellular energy charge, and an increase in lactate formation and catabolism of adenylates (Ronquist and Waldenström, 2003). These defects were accompanied by a significant decrease [relatively to both age-matched controls (AMC) and young adult controls (YC)] in the 2,3-DPG concentration that was accompanied by increases in the activity of diphosphoglycerate phosphatase (DPGP-ase), an enzyme that converts 2,3-DPG to 3PG (Kosenko et al., 2016). Of course, other factors besides of 2,3-DPG may affect the affinity of oxygen to hemoglobin (Samaja et al., 2003), but the relationship between the 2,3-DPG concentration in RBC as a biological indicator of tissue hypoxia in diabetic neuropathy (Nakamura et al., 1995), as well as in preterm infants with perinatal problems (Tsirka et al., 1990; Cholevas et al., 2008), in patients with the nondeletion genotype of hemoglobinopathy (Papassotiriou et al., 1998), with hypertension (Resnick et al., 1994), in experimental endotoxin shock (Matsumoto, 1995), severe hypophosphatemia (Larsen et al., 1996), and some types of glycolytic enzymes disturbances (McCully et al., 1999) was well established. Thus, the results generated the hypothesis that chronic enhancement in the rate of active transport in AD (Ronquist and Waldenström, 2003) leading to the increase in ATP and 2,3-DPG hydrolysis and can increase in Hb affinity to oxygen, loss of adequate oxygen delivery to tissues that may be one of the factors contributing to brain hypoxia (Aliev et al., 2004), glucose hypometabolism, and memory dysfunction in AD. It should be noted, however, that RBC of even cognitively stable aging persons (AMC) was characterized by a slight but significant decrease in 2,3-DPG when compared with the young adult control group. The tendency for the ATP production, adenylate energy charge, adenine nucleotide pool size, and ATP/ADP ratio (Kosenko et al., 2016) was a decrease in aging with no notable changes in dementia. There were no differences between AMC, AD, and NA groups in GSH levels, as well as in GSSG levels and the GSH/GSSG ratio in RBCs (Kosenko et al., 2012). Activities of calpain and caspase-3 in RBCs from aged subjects, on the contrary, were three times higher than those in young controls and were equally high in both dementia types (Kaminsky et al., 2012). The trend for the hydroperoxide generation was an increase in aging with no dramatic changes in dementia. There were no significant differences between AC, AD, and NA subjects in H_2_O_2_, organic hydroperoxide and the sum of H_2_O_2_ plus organic hydroperoxides content of RBC (Kaminsky et al., 2013). The results suggest that oxidative stress to some extent is already present in the RBC of the AMC subjects (Kosenko et al., 2012) and that together with the disturbances of glycolytic and transport processes and proteolysis increasing are a general feature of aging and not a feature of dementia. This view is supported by data comparing AD with normal aging, where was documented the same profile of damage (Smith et al., 1991; Moreira et al., 2006) suggesting that RBC oxidative damage is no longer an end stage but rather a signal of underlying changes of state (Moreira et al., 2006).

Although endogenous oxidative stress may damage the RBC itself the mass effect of large quantities of free radicals leaving the red cell has a prodigious potential to damage other components of the circulation (Johnson et al., 2005) including endothelial cells resulting in the microvascular pathology (Kiefmann et al., 2008). The combined effects of these damages most likely contribute to the morphological changes in oldster subjects (Richards et al., 2007), which may result in decreased erythrocyte deformability (Kuypers et al., 1990) and alter rheology and reduce the threshold for the development of neuropathology (Ajmani et al., 2003). We propose that the long-term lack of sufficient energy, disturbances of glycolytic pathway and sodium/potassium pump in aged subjects can decrease the ability of RBC to transfer oxygen, leading to inadequate tissue oxygenation and abnormal glucose metabolism in the brain and thereby reducing mental capacity and cognition. Thus, the reduced mental capacity may be, to a large extent, due to the imbalance in the metabolic processes in RBC. Obviously, other factors may be operative, but the role of RBC biochemical alterations as possible preclinical indicator of mental disorders must be critically examined. During the last 10 years, numerous biochemical abnormalities in RBC of subjects suffering from various mental disturbances have been detected (Danon et al., 1992; Rifkind et al., 1999; Ponizovsky et al., 2003; Pankowska et al., 2005; Lang et al., 2015; Pretorius et al., 2016). We believe that obligatory measurement of RBC biochemical parameters in peoples older than 50 years in the dynamics will help identify the risk factor for AD.

The problem is clear, but a number of questions arise in connection with the above-mentioned. If oxidative stress is more or less present in the erythrocytes of all elderly people and is a risk factor for dementia, why does this risk factor “work” for some people, while others, with the same risk factor, live to a very old age, maintaining “bright mind” and working capacity? The same question arises with regard to the concentration of 2,3-DPG reduction and the energy metabolism rate in the erythrocytes in general. It is obvious that the answers to these questions can only be obtained after identification of the reasons causing a global energy metabolism disorder, an increase of oxidative stress that are the basis of quick aging, affection of erythrocytes and that lead to a disruption of their functional capacity and early death. In other words, it is necessary to find out, under what influence factors (endogenous and exogenous) the reserve capacity of erythrocytes to withstand the stress that they are constantly exposed to, which circulate from the lungs to the tissues, decreases too soon.

Another problem is the lack of absolute knowledge of the hemopoiesis status in older people and especially in stressful situations that require intensification of the formation of blood cells. Numerous data indicate that the functions of basal hemopoiesis, which maintains the number of blood cells within the norm, changes insignificantly with age (Sansoni et al., 1993; Bagnara et al., 2000), whereas the reserve capacity of the bone marrow to resist stressful situations requiring its activation, even in healthy elderly people, reduces significantly with age (Williams et al., 1986; Globerson, 1999). For example, during bacterial infection or other periods of high hematopoietic demand, the formation of blood becomes “flaccid” and badly regulated, paradoxically (Rothstein, 1993), which makes it possible to assume that there is a hidden defect in the achievement of hematopoietic equilibrium in older people. Hence, the main question arises. Is such a hidden bone marrow defect typical for people with dementia? And does this defect lead only to the disruption of cellular equilibrium, or does it also cause the appearance of defective cells in the circulation that have not received adequate “strength reserve” in the bone marrow, and therefore they quickly age and get damaged in the bloodstream? It is clear that without the answers to these questions, it is impossible to evaluate the contribution of the impaired functional capacity of erythrocytes to the clinical symptoms of AD and other types of dementia. However, when dementia is exclusively referred to brain diseases, the attention of scientists is concentrated only on neurological symptoms, whereas all possible “defects” of erythrocytes, which cause pathological consequences for the brain, remain unexplored. We found only a few literature sources discussing the role of morphological changes that characterize the violation of the architecture of the erythrocyte membranes in the development of neurological symptoms characteristic of dementia (Mohanty et al., 2008). In particular, it has been shown that the appearance of atypical cells with altered morphological features, that is giant elongated erythrocytes with a nonhomogeneous membrane (acanthocytes or erythrocytes with numerous random spur-like cytoplasmic outgrowths) (Brecher and Bessis, 1972; Lan et al., 2015), occurs in advance (for several years) before the onset of memory disorders (Goodall et al., 1994). The mechanism of the acanthocytes emergence in the bloodstream is unknown, but since atypical cells are only a small part of the general population of normal erythrocytes, it has been suggested that the cause of their formation is related to a disruption in the synthesis of membrane structural proteins that occurs in the stage of erythrocyte formation in the bone marrow, although the possibility of the cell damage under the influence of unknown factors immediately after they come out from the bone marrow into the blood is not ruled out. Soluble amyloid peptides that are found in various cerebral vessels of the patients with AD [cerebral amyloid angiopathy (CAA)] and which, on the one hand, are capable to contact the cellular elements of blood, on the other—to damage the walls of blood vessels and cause a hemorrhage in the brain (Thanvi and Robinson, 2006), have recently been recognized as one of these factors. However, it is possible that the appearance of erythrocytes of the atypical form is associated with amyloids that circulate in the bloodstream and bind to the cell membrane (Kuo et al., 2000; Kiko et al., 2012) leading to its damage.

## The role of amyloid angiopathy in erythrocyte damage

The fact that AD is a systemic disease has been known for a long time, dating back to the last century, when amyloid peptides were first detected in small vessels of the brains of patients with AD (Scholz, 1938). For the sake of justice, it is worth mentioning that the existence of amyloid peptides was known back in 1878 when Atkins discovered amyloids in the brain and blood vessels of the brain in a young patient with dementia caused by a head injury (Atkins, 1878). At that time, it has been also identified that amyloids accumulate in the brain vessels in patients with syphilis (Atkins, 1875), and epilepsy (Blocq and Marinesco, 1892) indicating that cerebral amyloidosis and CAA are accompanying a number of diseases.

As it is now well known, in patients with DA, amyloids (mainly Aβ_1−40_) are found in the capillaries (Attems and Jellinger, 2004), arteries, arterioles, veins, and venules (Thal et al., 2002), which penetrate the leptomeningeal, cortical and subcortical areas of the brain (Weller et al., 2009), as well as in blood vessels supplying the hippocampus (Masuda et al., 1988). Localized in various structures of blood vessels (Wisniewski and Wegiel, 1994) and in contact with numerous cells (myocytes, pericytes), amyloids cause their damage (Vonsattel et al., 1991; Dalkara et al., 2011), as a result of which the membrane of the vessels seems to loosen, becoming unstable, which can eventually lead to the formation of an aneurysms, its rupture and cerebral hemorrhage (Thanvi and Robinson, 2006), that is, a condition that usually occurs with strokes and which irrespective of AD causes the formation of hematoma, lysis of erythrocytes, brain edema (Xi et al., 2006), local cell death, and memory damage (Pfeifer et al., 2002). Interestingly, the multiple microvascular pathology, mediated by the presence of amyloids in different structures of the blood vessels, was confirmed not only at postmortem examination, but also in life in virtually all patients with AD (Kalaria and Hedera, 1995; Farkas and Luiten, 2001; Bailey et al., 2004; Smith and Greenberg, 2009) regardless of the presence of atherosclerotic changes in the vessels. It should be noted, however, that CAA in AD patients is observed in 90–100% of cases, while brain zones with hemorrhage are detected only in 20–25% of patients with AD (Urbach, 2011). This means that brain damage in AD can occur for reasons not associated with CAA-induced hemorrhage. Indeed, a significant accumulation of amyloids in the brain vessels can cause their occlusion, thereby blocking blood flow, supplying the brain with oxygen and glucose (de la Torre and Stefano, 2000), and causing neurodegeneration of neurons and memory impairment (Thal et al., 2008, 2009). In addition, localized in endothelial cells lining the lumen of blood vessels (Michaud et al., 2013), amyloids are constantly in contact with erythrocytes circulating in the bloodstream, causing, on the one hand, their adhesion to endothelial cells, thereby violating the blood flow (Ravi et al., 2004), on the other—interacting with the membrane of erythrocytes, cause its modification and damage (Nicolay et al., 2007).

It was true that some studies have demonstrated that AD patients have increased RBC membrane injury suggesting the increased capability for erythrocyte lysis *in vivo* (Bosman et al., 1991; Goodall et al., 1994; Mattson et al., 1997; Solerte et al., 2000; Kosenko et al., 2009), as evidenced by the accumulation of free hemoglobin and iron in the brain of AD patients (Wu et al., 2004; Perry et al., 2008). The consequences of RBC lysis for the brain are well known (Xi et al., 1998). It has been shown that the appearance of free hemoglobin in the brain leads to rapid destruction of the hemato-encephalic barrier, DNA fragmentation, increased lipid peroxidation and global oxidative stress, development of the inflammatory process, vasoconstriction, hypoperfusion, brain atrophy (Alexander and LoVerme, 1980), memory impairment and death (Hackett and Anderson, 2000).

The lytic effect of amyloids was confirmed on the general population of erythrocytes. *In vitro* Aβ induces rapid lysis of human and rat erythrocytes that can be either attenuated by antioxidants (Mattson et al., 1997) or amplified in the presence of inhibitors of glycolytic and antioxidant enzymes or Na^+^, K^+^-ATPase (Kosenko et al., 2008a), and suggested the role of RBC glycolysis, ion pumping capacity and antioxidant status in the bioactivity and erythrotoxicity of amyloids. Given the above, we assumed that the constant contact of erythrocytes with amyloids can cause not only the change and damage on the membrane structures, but also the metabolic/energy metabolism in the erythrocytes underlying the aging, integrity, and functional ability of the cells. This assumption does not contradict the known pathological consequence of chronic brain hypoperfusion, leading to reducing oxygen delivery to the brain (de la Torre, 2017). On the contrary, it clearly points to the possible existence of additional unspecified mechanisms, restricting the oxygen supply to the brain and, therefore, participating in the development of hypoxia and neurodegenerative processes specific to AD (Thal et al., 2009). However, the population of erythrocytes is not homogeneous, and the facts that the least resistance of old erythrocytes to endogenous and exogenous pathological factors caused by a reduced rate of energy metabolism, antioxidant defense, and strengthening of catabolic processes (Bonsignore et al., 1964; Shinozuka et al., 1994) are well established.

**Figure 2:**
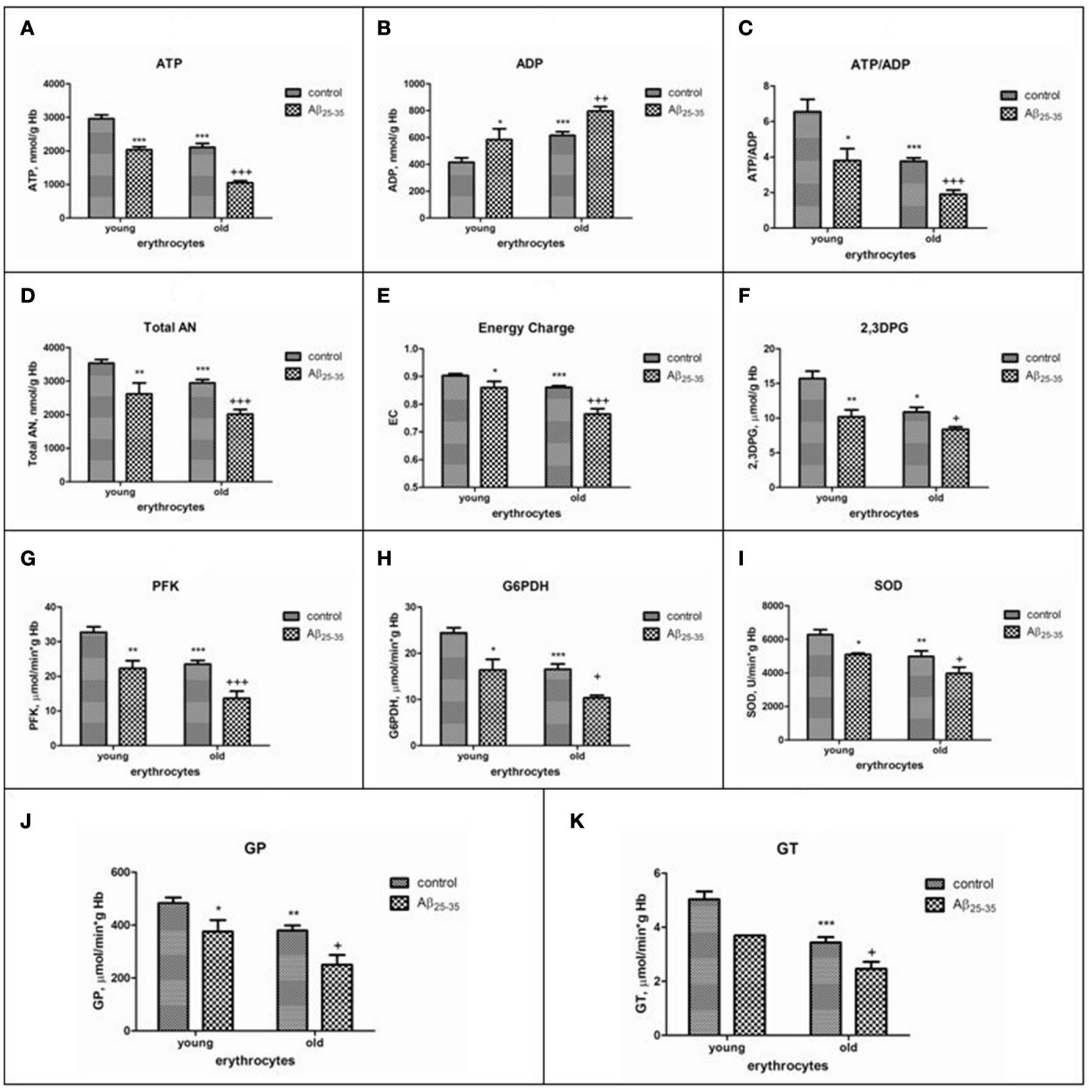
The effects of Aβ25-35 on the parameters of the adenylate system, concentration of 2,3–DPG and activities of some glycolytic and antioxidant enzymes activities in young and old erythrocytes (RBCs). **(A)** ATP, **(B)** ADP, **(C)** ratio ATP/ADP, **(D)** total adenine nucleotide pool size, **(E)** energy charge, **(F)** 2,3DPG, **(G–K)** activities of phosphofructokinase, glucose-6-phosphate dehydrogenase, superoxide dismutase, glutathione peroxidase, glutathione transferase, respectively. ATP and AN are expressed as micromol/g Hb, ADP as nmol/g Hb. AN, total adenine nucleotide pool size; EC, adenylate energy charge [EC = (ATP + 1/2ADP)/AN]; phosphofructokinase (PFK), glucose-6-phosphate dehydrogenase (G-6PDH), glutathione peroxidase (GP), glutathione transferase (GT) activities are expressed as IU/g hemoglobin (Hb); superoxide dismutase (SOD) is expressed as units/min per g Hb. One unit of SOD activity is defined as the amount of enzyme required to produce a 50% inhibition of the rate of p-nitrotetrazolium blue reduction. The results are the mean±SEM of 16 rats. Cells were incubated at 25°C for 30 min in 10 mmol/L potassium phosphate buffer, pH 7.4, containing 0.9% NaCl, 5 mmol/L KCl, and 10 μmol/L Aβ_25-35_. Control was incubated with nontoxic Aβ_35-25_. Significant differences are indicated: ^*^P < 0.05, ^**^P < 0.01, and ^***^P < 0.001 as compared to young cells; ^+^*P* < 0.05, ^++^*P* < 0.01, and ^+++^*P* < 0.001 as compared to the old control (one-way analysis of variance [ANOVA] with Bonferroni's multiple comparison test). Aβ indicates amyloid β.

Data on the effect of amyloid peptides on erythrocytes of different ages at present are currently not available and are of special interest, since patients with AD are characterized by accelerated aging of erythrocytes in the bloodstream (Bosman et al., 1991). We have recently showed that sensibility of RBC to βA-induced hemolysis was in proportion to both cell age and βA concentration (Tikhonova et al., 2014). The inhibition of glucose consumption and lactate production by βA was found to occur in both cells type. However, greater demand for ATP of the Na^+^, K^+^ -ATPase, in combination with a more reduced capacity of the glycolytic pathway and 2,3-DPG levels in old cells lead to more pronounced imbalance between ATP and 2,3-DPG formation, total nucleotide changes and ion pumping in aging erythrocytes exposed to the amyloid. Interestingly, the decline in the levels of antioxidative, glycolytic enzymes, 2,3DPG, ATP, adenine nucleotide pool and the adenylate energy charge in young cells treated with amyloid were similar to that we found as occurring during *in vivo* red cell aging (control old erythrocytes). Thus, our data obtained show that even a limited contact of amyloid with erythrocytes is sufficient to transform young erythrocytes into old ones and that similar biochemical mechanisms can underlie the accelerated aging of cells in the bloodstream of patients with AD (Bosman et al., 1991) (Figure 2), that may be one of the main reasons of both inadequate supply of oxygen to the brain, and lysis of cells in circulation.

## Novel therapeutic strategy: problems and possible solutions

At present there are no medical drugs which are able to increase and improve perfusion of the brain of AD patients, since due to the absence of early diagnostics the use of any drug therapy, when the brain tissue is irreparably damaged, is late and inefficient (Hachinski and Munoz, 1997). Thus, it is very problematic “to repair” chronically damaged blood vessels of the brain and to restore their functional state with the preparations available, as well as it is hard “to cure diseased erythrocytes.” This is connected, first of all, with the fact that real causes of “chronic disease” of erythrocytes during natural aging of the organism are unknown. One of the important problems, as noted above, is the absence of total knowledge on the hematopoietic status of the elderly, especially during long bed rest, accompanied by undernourishment, leads to a decrease in metabolism rate.

It is interesting to note that the problems in relation to the “disease” of erythrocytes arise during transfusion of the whole donor blood or packed RBC to the patients with different diseases in order to restore oxygen transport to the tissues and release carbonic acid from them. The main challenge is that all intracellular indices of erythrocytes change very quickly during the storage period of the donated blood leading to rapid cell aging (Lang et al., 2016). And if these indices are not corrected before blood transfusion this may result in irreparable consequences (Beutler et al., 1969). It has been shown, for example, that if during red blood cell transfusion intracellular ATP concentration of erythrocytes was lower by 40% compared to the normal cells, these erythrocytes were lysed to an excessive degree in blood flow of the recipient (Hamasaki et al., 1981). Transfusion of erythrocytes with low intracellular content of 2,3-DPG did not allow for quick restoration of adequate delivery of oxygen to the tissues. Taken together, these observations require development of the ways to increase the concentrations of ATP and 2,3-DPG in erythrocytes immediately before RBC transfusion to the patient (Beutler et al., 1969). Further studies in this field are actively undertaken, and multiple developments directed toward restoration of energy exchange in the stored erythrocytes are successively utilized by the physicians to save the patients life (Valeri and Hirsch, 1969). The main components restoring energy exchange in erythrocytes are glucose, adenine, ascorbate-2-phosphate, phosphoenolpyruvat or the cations and activators of glycolysis, which can penetrate into erythrocytes (Moore et al., 1981). It has been shown that different activators of enzymes introduced into the medium, where erythrocytes are stored, maintain normal concentration of ATP and 2,3-DPG for 1,5 months (Vora, 1987). However, although the scientists have made a considerable progress in solving the problems with regard to restoration of energy exchange disturbed during storage of erythrocytes, all the developments use modulators and activators, which are able to quickly and easily pass through the cell membrane of erythrocytes. This is a limitation for the use of the wider class of active compounds that are unable to be transported into the cells. We tried to circumvent this problem and developed a technology of the encapsulation of substrates and high molecular enzymes in erythrocytes under hypotonic conditions leading to the formation of pores in the membranes of erythrocytes (Seeman et al., 1973), enabling the enzymes with great molecular mass (Baker, 1967; Kosenko et al., 2008b; Godfrin et al., 2012; Kaminsky and Kosenko, 2012; Alexandrovich et al., 2017) to pass through the cells. For instance, we developed an approach on how to introduce regulatory glycolytic enzymes into erythrocytes, where the activity of these enzymes in erythrocytes of old animals and in the elderly decreased by 30–50% (Kaminsky et al., 2013). The data obtained showed that the encapsulation of even one regulatory enzyme in erythrocytes stimulated glycolysis to considerable extent. The signs of it were the increased rate of glucose consumption and the formation of lactate. Moreover, the erythrocytes obtained circulated in the animal's blood flow within many days, sustaining the activity of encapsulated enzymes, the normal level of ATP, 2,3-DPG and other metabolites of energy exchange and antioxidant defense (data not shown). These results obtained are important by two reasons. First, such technology can be applied to restore ATP, 2,3-DPG and other metabolites of energy exchange, the concentration of which is decreased sharply in erythrocytes in aging of the organism. Transfusion of own erythrocytes, possessing encapsulated enzymes, should theoretically reduce the risk of the onset of inadequate oxygen supply to the brain both in the elderly and in patients with AD. Secondly, investigations make the basis for further development of innovative personalized therapeutic strategy.

## Conclusions

Presently, non-genetic Alzheimer's disease is classified as a neurodegenerative disorder. However, there is an impressive body of evidence indicating that AD is a systemic metabolic disease (Perry et al., 2003), and it has originated as a vascular disorder with the resultant impairment of the delivery and transport of essential nutrients, particularly glucose and oxygen resulting in an energy metabolic breakdown with the plaques and tangles found in the brain secondary to the effects of the vascular pathology (de la Torre, 2002a). Since erythrocyte serve as the only oxygen carrier and their ability to the binding, transport, and delivery of oxygen to tissues depends, first of all, on the energy metabolism and antioxidant status there is therefore a strong possibility that the disturbance of energy metabolism and oxidative enhancement in these cells may have a dramatic impact on destabilization of aerobic glucose metabolism in the brain and AD development. With regard to RBC-controlled brain vital activity there is incontrovertible evidence that even just a few minutes of oxygen deprivation (together with glucose) initiate significant brain dysfunction and chronic effect can ultimately result in the irreversible brain damage and permanent impairment of cognition. This implies that the cerebrometabolic abnormalities are the most common form of dementia (Chibber et al., 2016; Gonzalez-Reyes et al., 2016). However, although major mechanisms involved in brain damage due to metabolic abnormalities resulting from the oxygen deprivation including alterations in neurotransmission, defect of mitochondrial oxidative phosphorylation, disturbance of Ca^2+^ homeostasis, oxidative stress and eventually apoptotic or necrotic cell death are profound and obvious (Barreto et al., 2011; Cabezas et al., 2012, 2015; Avila Rodriguez et al., 2014; Toro-Urrego et al., 2016; Baez et al., 2017; Baez-Jurado et al., 2017a,b; Martin-Jiménez et al., 2017a,b; Shevtsova et al., 2017), no systematic programs of research have examined the relationship between the breach of the energy metabolism of erythrocytes in the causing of leading to cerebrometabolic abnormalities and dementia. One of the reasons of this paradox is the large number of reports stating that brain atrophy and degeneration of nerve cells, observed with dementia, can occur without cerebrovascular pathology, but only through the amyloid fault, leading to the struggle with amyloids, and not with the causes that “gave birth to them,” and made the AD a permanently incurable disease with unknown etiology. In our view, a careful examination and reversing age-related metabolic/energetic changes in erythrocytes is an achievable goal and will provide these cells as a marker of a risk of inadequate brain oxygen supply, resulting the irreversible brain damage and permanent impairment of cognition. We also strongly believe that biochemical erythrocyte indicators (ATP, 2,3DPG, glucose, lactate and others), as well as the enzymes of glycolysis, pentose phosphate and Rapoport-Luebering shunt, antioxidant pathways all of which are responsible for interrelated metabolism and functional capacity of RBC should be studied (especially in people over 50 years, and in the dynamics) not only in research laboratories, but also in clinical settings that may provide a basis for innovative personalized therapeutic strategies.

The development of technologies to assist in restoration of erythrocyte energy metabolism must form an integral part of new therapeutic strategies in the treatment of a great variety of disorders accompanied by inadequate oxygen delivery. Similar studies just are gathering pace but have already marked a turning-point in our knowledge regarding AD and amyloid peptides that cannot be the only pharmacological target in the struggle against this devastating illness of human beings.

## Author contributions

All of authors (EK, LT, CM, GB, GA, and YK) write manuscript, created figures, and proof final version of this manuscript.

### Conflict of interest statement

The authors declare that the research was conducted in the absence of any commercial or financial relationships that could be construed as a potential conflict of interest.
